# Investigating Unhealthy Alcohol Use As an Independent Risk Factor for Increased COVID-19 Disease Severity: Observational Cross-sectional Study

**DOI:** 10.2196/33022

**Published:** 2021-11-05

**Authors:** Sameer Bhalla, Brihat Sharma, Dale Smith, Randy Boley, Connor McCluskey, Yousaf Ilyas, Majid Afshar, Robert Balk, Niranjan Karnik, Ali Keshavarzian

**Affiliations:** 1 Department of Internal Medicine Rush University Medical Center Chicago, IL United States; 2 Addiction Data Science Laboratory Department of Psychiatry & Behavioral Sciences Rush University Medical Center Chicago, IL United States; 3 Department of Medicine School of Medicine and Public Health University of Wisconsin Madison, WI United States; 4 Center for Integrated Microbiome and Chronobiology Research Rush University Medical Center Chicago, IL United States; 5 Center for Circadian Rhythm and Alcohol-Induced Tissue Injury Rush University Medical Center Chicago, IL United States

**Keywords:** unhealthy alcohol use, COVID-19, SARS-CoV-2, acute respiratory distress syndrome, substance misuse, mechanical ventilation, substance use

## Abstract

**Background:**

Unhealthy alcohol use (UAU) is known to disrupt pulmonary immune mechanisms and increase the risk of acute respiratory distress syndrome in patients with pneumonia; however, little is known about the effects of UAU on outcomes in patients with COVID-19 pneumonia. To our knowledge, this is the first observational cross-sectional study that aims to understand the effect of UAU on the severity of COVID-19.

**Objective:**

We aim to determine if UAU is associated with more severe clinical presentation and worse health outcomes related to COVID-19 and if socioeconomic status, smoking, age, BMI, race/ethnicity, and pattern of alcohol use modify the risk.

**Methods:**

In this observational cross-sectional study that took place between January 1, 2020, and December 31, 2020, we ran a digital machine learning classifier on the electronic health record of patients who tested positive for SARS-CoV-2 via nasopharyngeal swab or had two COVID-19 International Classification of Disease, 10th Revision (ICD-10) codes to identify patients with UAU. After controlling for age, sex, ethnicity, BMI, smoking status, insurance status, and presence of ICD-10 codes for cancer, cardiovascular disease, and diabetes, we then performed a multivariable regression to examine the relationship between UAU and COVID-19 severity as measured by hospital care level (ie, emergency department admission, emergency department admission with ventilator, or death). We used a predefined cutoff with optimal sensitivity and specificity on the digital classifier to compare disease severity in patients with and without UAU. Models were adjusted for age, sex, race/ethnicity, BMI, smoking status, and insurance status.

**Results:**

Each incremental increase in the predicted probability from the digital alcohol classifier was associated with a greater odds risk for more severe COVID-19 disease (odds ratio 1.15, 95% CI 1.10-1.20). We found that patients in the unhealthy alcohol group had a greater odds risk to develop more severe disease (odds ratio 1.89, 95% CI 1.17-3.06), suggesting that UAU was associated with an 89% increase in the odds of being in a higher severity category.

**Conclusions:**

In patients infected with SARS-CoV-2, UAU is an independent risk factor associated with greater disease severity and/or death.

## Introduction

In patients with COVID-19, age, obesity, smoking, and chronic comorbidities are risk factors that impact the rate of contracting COVID-19 and severity of infection; however, a significant number of patients without these comorbidities also develop severe disease [1,2]. This suggests that additional risk factors may promote an exaggerated immune response to the virus. Alcohol is the most common drug used in the United States and its use has increased during the COVID-19 pandemic [3,4]. Unhealthy alcohol use (UAU) is known to interrupt pulmonary immune mechanisms and can lead to increased rates of viral pneumonia and progression into acute respiratory distress syndrome (ARDS) [5]. Chronic alcohol consumption also causes severe oxidative stress that may lead to an increased susceptibility for sepsis-mediated ARDS [6]. Despite the known deleterious effect of alcohol use on the pulmonary immune system, many of the early large studies performed on patients with COVID-19 did not include alcohol use history [7]. Furthermore, a meta-analysis of six studies found that alcohol use did not impact the severity of COVID-19 infection [8]. The studies described in this meta-analysis all originate from China, and among the major limitations is a purely clinical assessment of UAU (without validated measures). Another prospective cohort study in the United Kingdom examined the relationship between lifestyle risk factors (physical inactivity, smoking, obesity, and UAU) and COVID-19 infection and found that UAU was not related to COVID-19 disease [9]. A more recent study in the United States suggested that the alcohol lung represents a very likely comorbidity for the negative consequences of both COVID-19 susceptibility and severity [10]. Generally, research on alcohol and substance use has been limited by small samples with the use of validated measures or large samples reliant on International Classification of Disease, 10th Revision (ICD-10) codes. The latter is believed to severely underreport UAU and substance misuse. Due to the sparse evidence and conflicting theories, we aim to further study the interaction between UAU and COVID-19 disease severity using a novel approach to identify UAU and we believe that these results will better inform treatment management of at-risk patients.

## Methods

### Recruitment

Males and females aged ≥18 years who tested positive for SARS-CoV-2 via a nasopharyngeal swab or had two COVID-19 ICD-10 codes at Rush University Medical Center between January 1, 2020, and December 31, 2020, were included in this observational cross-sectional study. Patients younger than 18 years of age were excluded from the study. This study was approved by the Rush University Medical Center Institutional Review Board (17090601-IRB02) and informed consent was waived. Demographic data were extracted from the electronic health record (EHR) including sex, age, BMI, and race/ethnicity (Table 1). Each patient’s COVID-19 severity was determined by the maximal level of care they received. COVID-19 severity categories were defined as the following: (1) emergency department admission without need for a ventilator; (2) emergency department admission requiring use of a ventilator; and (3) death.

To identify cases of UAU, we used a digital machine learning classifier that was applied to all clinical notes in the EHR [11]. Free-text clinical notes containing details about a patient’s behavioral conditions were used to feed the alcohol misuse digital classifier with methods of natural language processing with supervised machine learning to predict a patient’s probability of UAU. The classifier has demonstrated excellent ability to predict alcohol misuse based on validation against ICD-10 codes as well as manual annotation of charts. A study that is presently under review has also found that the classifier is accurate, using the Alcohol Use Disorders Identification Test (AUDIT) as the reference standard. A higher predictive probability was previously shown to indicate a greater likelihood of UAU and had a dose-dependent correlation with severity of UAU [11]. For analysis, the predicted probabilities were log-transformed to account for their non-normal distribution. The probability of having UAU as determined by the classifier was entered into the model examining the association with the primary outcome.

### Statistical Analysis

To account for patients with repeat hospital encounters, we performed mixed effects ordinal logistic regression analysis with random intercepts to predict a patient’s COVID-19 severity group. Additionally, we performed two sensitivity analyses to assess the robustness of the classifier in predicting outcome severity across different parameterizations. In the first analysis, patients were categorized into alcohol/nonalcohol groups and the mixed effects ordinal logistic regression was used to predict COVID-19 severity. In the second analysis, severity outcome was dichotomized into two categories to represent severity of disease by hospital disposition: (1) emergency department admission without requiring a ventilator and (2) emergency department admission requiring the use of a ventilator or death. All analyses controlled for age, sex, ethnicity, BMI, smoking status, insurance status, and presence of ICD-10 codes for cancer, cardiovascular disease, and diabetes. We further explored interactions between the classifier and these demographic characteristics to assess potential moderation by these variables. All analyses were conducted using a significance level of .05 in Python (version 3.9.0; Python Software Foundation) and Stata (version 17; StataCorp LLC).

## Results

**Figure 1:**
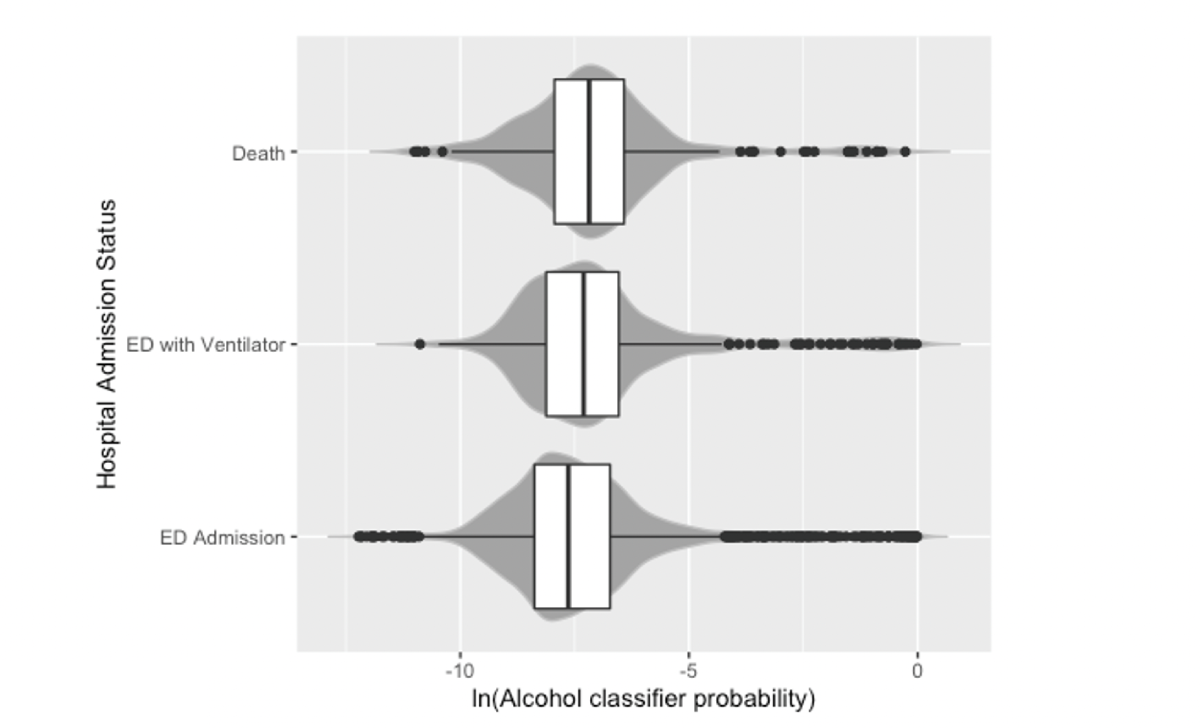
Increased likelihood of unhealthy alcohol use (determined by a digital machine learning classifier) in patients diagnosed with COVID-19 at a large academic hospital in Chicago, Illinois, between January 1, 2020, and December 31, 2020, was associated with more severe disease outcomes as measured by hospital admission status (odds ratio 1.24, 95% CI 1.14-1.37; *P*<.001). ED: emergency department.

**Figure 2:**
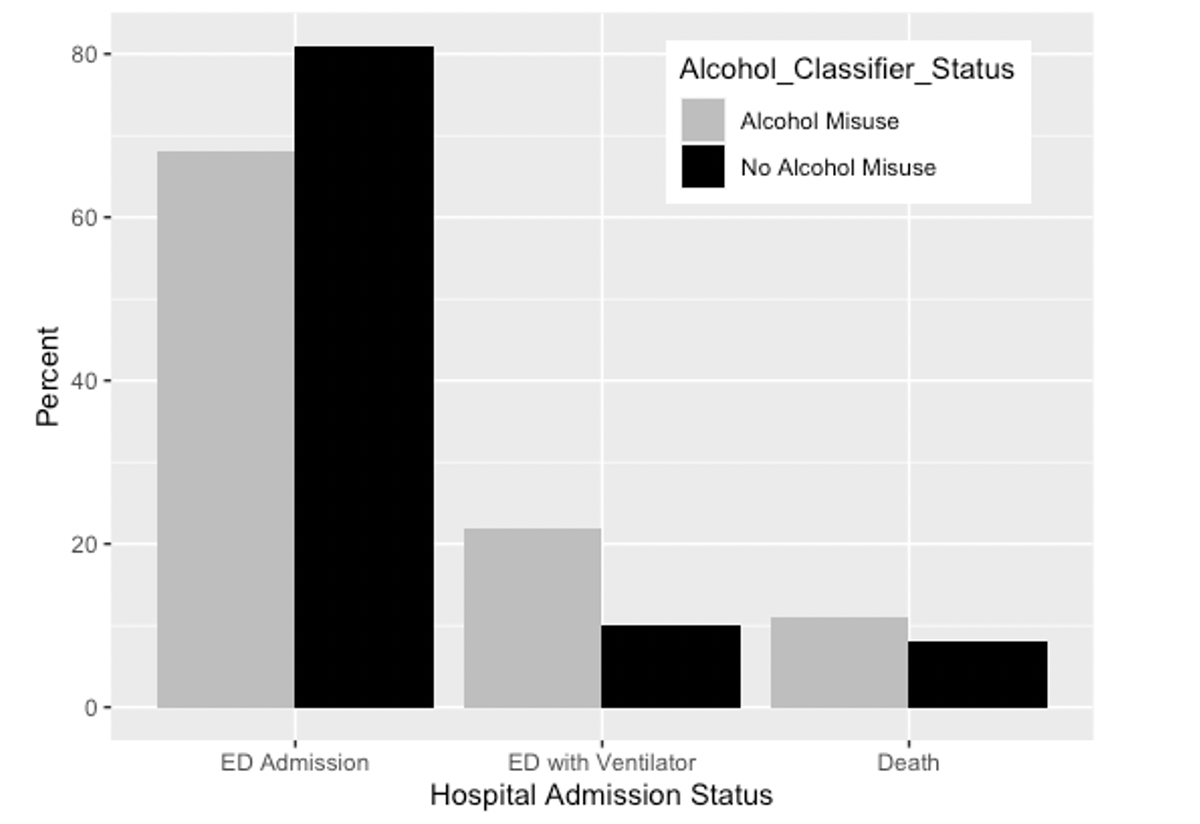
Hospital admission status for 3480 patients in Chicago, Illinois, who were diagnosed with COVID-19 at a large academic hospital between January 1, 2020, and December 31, 2020, stratified by their alcohol misuse status determined by a digital machine learning classifier for alcohol misuse. ED: emergency department.

In total, 3480 patients, who accounted for 4134 hospital encounters, were included for analysis. Overall patient characteristics are depicted in Table 1. We found that as the probability of predicting UAU increases, so does the risk for poor health outcomes (odds ratio 1.24, 95% CI 1.14-1.37; Figure 1). Age, sex, and BMI were also associated with COVID-19 severity status, but smoking status, ethnicity, insurance status, or presence of other health condition codes were not (Table 2). No interactions between classifier status and demographic variables were significant (*P*>.40). When we dichotomized patients’ classifier status into those with UAU and those without, we found that this dichotomous classification was also associated with COVID-19 severity (odds ratio 1.85, 95% CI 1.11-3.09; see Table S1 in Multimedia Appendix 1). The distribution of hospital admission type stratified by patients’ alcohol misuse status is depicted in Figure 2. Of the patients with alcohol misuse, 67.8% (61/90) were inpatient admissions from the emergency department (ED), 22.2% (20/90) were admitted through the ED and required a ventilator during their hospitalization, and 10.0% (9/90) died (Figure 2). Of the patients with no alcohol misuse, 81.4% (3292/4042) were inpatient ED admissions, 10.8% (437/4042) were admitted through the ED and required a ventilator during their hospitalization, and 7.8% (313/4042) died (Figure 2). The ability for alcohol classifier status to predict COVID-19 severity was also robust to reparameterization of severity into two severity categories, as the alcohol classifier estimate was associated with increased odds of ventilation or death in the dichotomous outcome model (odds ratio 1.15, 95% CI 1.09-1.22; see Table S2 in Multimedia Appendix 1).

**Table 1:** Patient characteristics from an observational cross-sectional study of patients diagnosed with COVID-19 conducted in Chicago, Illinois, between January 1, 2020, and December 31, 2020, investigating the relationship between alcohol use and COVID-19 disease severity.

Demographics		Values
Age (years), mean (SD)		59.15 (17.52)
**Sex, n (%)**		
	Male	1793 (51.52)
	Female	1687 (48.48)
**Race/ethnicity, n (%)**		
	Non-Hispanic White	713 (20.49)
	Non-Hispanic Black	1447 (41.58)
	Hispanic	1010 (29.02)
	Other	310 (8.91)
BMI, mean (SD)		31.62 (10.18)
**Smoking status, n (%)**		
	Never smoker	1631 (61.22)
	Quit	815 (30.59)
	Current smoker (some days)	61 (2.29)
	Current smoker (every day)	157 (5.89)
Length of hospital stay (days), mean (SD)		8.39 (9.28)
Minimum oxygen saturation (%), mean (SD)		81.04 (18.34)
**Insurance status, n (%)**		
	Medicaid	1268 (36.44)
	Medicare	1099 (31.58)
	Private	817 (23.48)
	Other	296 (8.51)
**International Classification of Disease, n (%)**		
	Cancer	117 (3.36)
	Cardiovascular	393 (11.29)
	Diabetes	215 (6.18)

**Table 2:** Adjusted associations between risk factors and severity of COVID-19 in patients diagnosed with COVID-19 at a large academic hospital in Chicago, Illinois, between January 1, 2020, and December 31, 2020 (N=4134).

Predictor		Unhealthy alcohol use	No unhealthy alcohol use	Odds ratio		95% CI		*P* value	
Alcohol classifier, n		90	4044	1.24		1.14-1.37		<.001	
Age		N/A^a^	N/A	1.02		1.01-1.03		<.001	
Mean age (SD), years		59.58 (17.29)	50.12 (18.07)	N/A		N/A			
**Sex, n**					0.64		0.49-0.85		.002
	Male	66	2050	N/A		N/A			
	Female	24	1994	N/A		N/A			
**Race/ethnicity, n**									
	Non-Hispanic White	18	823	Reference					
	Non-Hispanic Black	42	1715	0.95		0.68-1.35		.79	
	Hispanic	24	1171	1.17		0.81-1.70		.41	
	Other	6	335	1.46		0.88-2.41		.14	
BMI, mean (SD)		31.71 (10.20)	26.93 (7.73)	1.02		1.01-1.04		.001	
**Smoking status, n**									
	Never smoker	22	1885	Reference					
	Quit	18	1001	0.85		0.67-1.08		.25	
	Current smoker (some days)	6	68	0.59		0.27-1.27		.07	
	Current smoker (every day)	17	161	1.14		0.75-1.75		.13	
**Insurance status, n**									
	Medicaid	51	1429	Reference					
	Medicare	11	1387	0.83		0.60-1.15		.25	
	Private	10	909	0.73		0.52-1.03		.07	
	Other	18	319	0.68		0.41-1.11		.13	
**International Classification of Disease, n**									
	Cancer	0	142	0.49		0.22-1.08		.08	
	Cardiovascular	3	476	0.76		0.51-1.13		.17	
	Diabetes	1	265	1.1		0.68-1.77		.70	

^a^N/A: not applicable.

## Discussion

### Principal Results

Alcohol interferes with pulmonary adaptive and innate immunity, increases susceptibility to viral infections, and increases the risk for developing ARDS [5,12]. Furthermore, alcohol is exhaled by the lungs, and a patient with impaired lung function due to COVID-19 pneumonia could have increased levels of toxic alcohol metabolites in addition to compromised defense mechanisms. To test our hypothesis that alcohol use increases the risk for severe COVID-19 infection, we used a previously proven digital machine learning classifier to predict patients’ alcohol use and severity and found an association between the predicted probability of alcohol use and COVID-19 disease severity. The results also suggest that UAU classification was associated with a nearly two-fold odds risk of being in a higher COVID-19 disease severity category.

### Comparison With Prior Work

There is limited data regarding risks, disparity, and outcomes for COVID-19 in individuals with substance use disorders. Prior research demonstrated that patients diagnosed with substance misuse disorder in the 12 months before contracting COVID-19 had increased rates of hospitalization, ventilator use, and mortality [13,14]. Only one of these studies differentiated based on type of substance misuse and found the risk for those with opioid misuse to be the highest, followed by those with tobacco use disorder, then alcohol use disorder [14]. Our study further explores the effect UAU has on COVID-19 disease outcomes. Our findings are supported by prior studies that suggest that alcohol exposure may augment the activity of proinflammatory cytokines (interleukin 1β, interleukin 6, and tumor necrosis factor) in SARS-CoV-2 infection and cause pulmonary, gastrointestinal, hepatic, and neurologic organ dysfunction [15,16]. Another study demonstrated that alcohol causes severe oxidative stress due to the depletion of glutathione in the alveolar space, which leads to increased susceptibility to sepsis-mediated ARDS [17]. Our study contradicts the meta-analysis of 6 Chinese studies that found alcohol consumption did not affect COVID-19 disease severity [8]. This is likely due to our larger sample size and heterogenous population, but could also be explained by differing cultural definitions of UAU. Nonetheless, further studies must be conducted to understand the exact mechanisms linking alcohol consumption and COVID-19 disease severity.

### Limitations

Our study is not without limitations. The machine learning classifier is not a diagnostic tool; rather, it is a probability predictor of UAU. More information on quantity and frequency of alcohol use would be helpful in parsing out a dose-dependent effect of alcohol on COVID-19 disease severity. However, our classifier provides a practical tool to screen for UAU using the first 24 hours of EHR notes collected during usual care. We previously showed good screening characteristics and anticipate that as natural language processing becomes more commonplace, these tools can be deployed to help physicians intervene on a modifiable risk factor like UAU. Another limitation is that there may also be other factors that contributed to the severity of COVID-19 disease that are not adequately captured in the EHR data. This study was meant to be a preliminary examination; therefore, we only examined three comorbid conditions. There are several others identified by the Centers for Disease Control and Prevention that could be included in future studies regarding COVID-19 disease severity. Our data also does not take into account the effect vaccines have on COVID-19 disease severity in those who have UAU. Future research that examines disease severity in vaccinated adults with UAU may be of interest.

### Conclusions

Using a previously tested machine learning classifier for UAU, we studied the effect alcohol may have on COVID-19 disease severity. We found that those who were more likely to practice UAU were significantly more likely to require a ventilator and die if they contracted COVID-19. Therefore, we concluded that UAU is an independent risk factor for more severe COVID-19 disease. As the risk for COVID-19 infection persists, providers should be mindful of vulnerable patient populations that may be more likely to experience severe disease, and attempt to encourage patients to get vaccinated and reduce their alcohol use.

## Appendix

Multimedia Appendix 1 Supplemental data.

## Abbreviations

ARDS: acute respiratory distress syndrome
AUDIT: Alcohol Use Disorders Identification Test
ED: emergency department
EHR: electronic health record
ICD-10: International Classification of Disease, 10th Revision
UAU: unhealthy alcohol use
