# Supplementary material for: Investigating Unhealthy Alcohol Use As an Independent Risk Factor for Increased COVID-19 Disease Severity: Observational Cross-sectional Study
Source: JMIR Public Health Surveill. 2021 Nov 5;7(11):e33022. doi: 10.2196/33022 (PMC8575002; doi:10.2196/33022)
Supplement: Multimedia Appendix 1 [file publichealth_v7i11e33022_app1.docx]

Multimedia Appendix 1.

| **Predictor** | **Odds Ratio** | **95% Confidence Interval** | **P** |
| --- | --- | --- | --- |
| Alcohol Classifier^1^ | 1.85 | 1.11 – 3.09 | 0.019 |
| Age | 1.01 | 1.00 – 1.02 | 0.005 |
| Sex | 0.68 | 0.57 – 0.82 | < 0.001 |
| Race/Ethnicity |  |  |  |
| Non-Hispanic White | Ref. |  |  |
| Non-Hispanic Black | 0.91 | 0.72 – 1.16 | 0.45 |
| Hispanic | 1.16 | 0.90 – 1.51 | 0.257 |
| Other | 1.29 | 0.91 – 1.82 | 0.146 |
| Body Mass Index | 1.01 | 1.00 – 1.02 | 0.003 |
| Smoking Status |  |  |  |
| Never Smoker | Ref. |  |  |
| Quit | 0.91 | 0.72 – 1.15 | 0.44 |
| Current Smoker (some days) | 0.66 | 0.31 – 1.41 | 0.284 |
| Current Smoker (every day) | 1.36 | 0.91 – 2.05 | 0.138 |
| Insurance Status |  |  |  |
| Medicaid | Ref. |  |  |
| Medicare | 0.84 | 0.67 – 1.04 | 0.113 |
| Private | 0.79 | 0.62 – 1.00 | 0.048 |
| Other | 0.77 | 0.55 – 1.10 | 0.151 |
| Comorbid Condition |  |  |  |
| Cancer | 0.58 | 0.32 – 1.04 | 0.068 |
| Cardiovascular | 0.83 | 0.63 – 1.11 | 0.207 |
| Diabetes | 1.02 | 0.73 – 1.44 | 0.898 |

*Table S1:* ^1^A cut-off of 0.15 on the digital aclohol classifier was used to classify patients with unhealthy alcohol use. The table depicts adjusted associations between risk factor and severity of COVID-19 disease in patients diagnosed with COVID-19 at a large academic hospital in Chicago, Illinois between January 1, 2020 and December 31, 2020 (Odds ratios and 95% confidence intervals) n = 3,480.

| **Predictor** | **Odds Ratio** | **95% Confidence Interval** | **P** |
| --- | --- | --- | --- |
| Alcohol Classifier | 1.15 | 1.09, 1.22 | < 0.001 |
| Age | 1.01 | 1.00, 1.02 | 0.001 |
| Sex | 0.72 | 0.60, 0.87 | 0.001 |
| Race/Ethnicity |  |  |  |
| Non-Hispanic White | Ref. |  |  |
| Non-Hispanic Black | 0.91 | 0.71, 1.16 | 0.435 |
| Hispanic | 1.15 | 0.88, 1.48 | 0.332 |
| Other | 1.25 | 0.88, 1.77 | 0.214 |
| Body Mass Index | 1.01 | 1.00, 1.02 | 0.003 |

*Table S2:* The digital alcohol classifier was able to predict COVID-19 severity following reparameterization of outcomes into two severity categories (emergency department admission without requiring a ventilator and emergency department admission requiring use of a ventilator or death). The study investigates how likelihood of alcohol misuse impacts COVID-19 disease severity in patients diagnosed with COVID-19 between January 1, 2020 and Decemeber 31, 2020 at a larger academic hospital in Chicago, Illinois.
